# Long-Term Measurements of Radon and Thoron Exhalation Rates from the Ground Using the Vertical Distributions of Their Activity Concentrations

**DOI:** 10.3390/ijerph18041489

**Published:** 2021-02-04

**Authors:** Oumar Bobbo Modibo, Yuki Tamakuma, Takahito Suzuki, Ryohei Yamada, Weihai Zhuo, Chutima Kranrod, Kazuki Iwaoka, Naofumi Akata, Masahiro Hosoda, Shinji Tokonami

**Affiliations:** 1 Department of Radiation Science, Graduate School of Health Sciences, Hirosaki University, 66-1 Honcho, Hirosaki, Aomori 036-8564, Japan; h19gg701@hirosaki-u.ac.jp (O.B.M.); tamakuma@hirosaki-u.ac.jp (Y.T.); suzuki-takahito@fujielectric.com (T.S.); yamada.ryohei@jaea.go.jp (R.Y.); m_hosoda@hirosaki-u.ac.jp (M.H.); 2 Nuclear Technology Section, Institute of Geological and Mining Research, P.O. Box 4110, Yaoundé, Cameroon; 3 Institute of Radiation Emergency Medicine, Hirosaki University, 66-1 Honcho, Hirosaki, Aomori 036-8564, Japan; kranrodc@hirosaki-u.ac.jp (C.K.); akata@hirosaki-u.ac.jp (N.A.); 4 Institute of Radiation Medicine, Fudan University, 2094 Xietu Road, Shanghai 200032, China; whzhuo@fudan.edu.cn; 5 National Institutes for Quantum and Radiological Science and Technology, 4-9-1 Anagawa, Inage, Chiba 263-0024, Japan; iwaoka.kazuki@qst.go.jp

**Keywords:** exhalation rate, radon, thoron, long-term measurement, seasonal variation

## Abstract

A long-term measurement technique of radon exhalation rate was previously developed using a passive type radon and thoron discriminative monitor and a ventilated type accumulation chamber. In the present study, this technique was applied to evaluate the thoron exhalation rate as well, and long-term measurements of radon and thoron exhalation rates were conducted for four years in Gifu Prefecture. The ventilated type accumulation chamber (0.8 × 0.8 × 1.0 m^3^) with an open bottom was embedded 15 cm into the ground. The vertical distributions of radon and thoron activity concentrations from the ground were obtained using passive type radon-thoron discriminative monitors (RADUETs). The RADUETs were placed at 1, 3, 10, 30, and 80 cm above the ground inside the accumulation chamber. The measurements were conducted from autumn 2014 to autumn 2018. These long-term results were found to be in good agreement with the values obtained by another methodology. The radon exhalation rates from the ground showed a clearly seasonal variation. Similar to findings of previous studies, radon exhalation rates from summer to autumn were relatively higher than those from winter to spring. In contrast, thoron exhalation rates were not found to show seasonal variation.

## 1. Introduction

Radon (^222^Rn) and thoron (^220^Rn) are naturally occurring radioactive gases generated from the ^238^U- and ^232^Th-series. It is well known that radon and thoron are the biggest contributors to human radiation exposure from natural sources [1]. The World Health Organization (WHO) has recognized them as the second largest cause of lung cancer after smoking [2]. Indoor and outdoor radon and thoron concentrations vary widely from place to place depending on geological features and meteorological condition of an area (see, e.g., [3]). In general, indoor radon concentration is continuously supplied by a portion of outdoor radon, an infiltration rate of 10 Bq m^−3^ h^−1^ was reported [1]. In addition to the health effect assessment due to its inhalation, outdoor radon monitoring is useful in several scientific disciplines as a radioactive tracer. Its half-life of T_1/2_ = 3.82 days is comparable to the air masses’ transit time across the major continents. Outdoor radon monitoring serves also on earthquake forecasting, geological faults identifications or ore exploration, and environmental reprocessing in mining [4,5,6,7]. Some researchers have reported a positive correlation between outdoor radon concentration and radon exhalation rate from the ground [8,9,10]. Therefore, the exhalation rates of radon and thoron, which are often called flux or flux density, are useful parameters to understand human health risk due to radon and thoron inhalation, and many researchers have reported data obtained by field and experimental studies [11,12,13,14]. Generally, a common technique for exhalation rate measurement is based on placing an accumulation chamber on the ground surface to accumulate radon gas exhaling from the ground and using radon monitor to measure radon concentration and deduce radon exhalation rate, the technique has been applied for short- and long-term radon exhalation rate measurements [15]. However, it is difficult to evaluate both radon and thoron exhalation rates simultaneously using this method due to short half-life of thoron (T_1/2_ = 55.6 s). Alternatively, Zhuo et al. [10] reported on the long-term measurement technique of radon exhalation rate using a passive type radon and thoron discriminative monitor and a ventilated type accumulation chamber. However, their report did not evaluate thoron exhalation rate. It was reported that thoron activity concentrations from a source such as the materials of building walls and the ground have a unique distribution [16,17,18,19]. In the present study, long-term radon and thoron exhalation rates from the ground were simultaneously measured for a period of four years by applying the previously reported technique of ventilated type accumulation chamber [9]. From the results obtained, the seasonal variations of radon and thoron exhalation rates from the ground were discussed.

## 2. Materials and Methods 

### 2.1. Ventilated-Type Accumulation Chamber System for Measuring Radon-Thoron Exhalation Rates from the Ground

**Figure 1:**
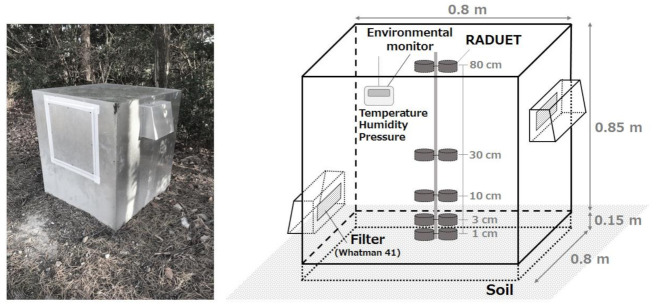
Photo and schematic drawing of the ventilation-type accumulation chamber system for measuring radon and thoron exhalation rates from the ground.

A naturally ventilated accumulation chamber (0.8 × 0.8 × 1.0 m^3^) which is a stainless-steel box with an open bottom was embedded 15 cm into the ground on the campus of the National Institute of Fusion Science (NIFS) located in Gifu Prefecture, Japan (N35.325°, E137.168°), as shown in Figure 1. Two rectangle openings (20 × 10 cm^2^) were perforated at the upper right and lower left walls of the stainless-steel box to get air ventilation, and each opening was covered on the inside side by a fiber filter (Whatman^®^ No. 41) and the outside side by a rain/wind shelter. Thus, the change of particles (dust) outside cannot interfere with the inside environment of the stainless-steel box as the two openings are covered with filters. According to the report by Zhuo et al. [10], wind speed inside and outside of the ventilated accumulation chamber was monitored, and it was found that the inside wind speed could hardly be affected by the change of outside winds. Additionally, the air and soil conditions (pressure, temperature, relative humidity, and water potential) monitored simultaneously inside and outside of the stainless-steel box showed that except for the air humidity both the soil and air conditions inside and outside were nearly the same throughout the year, and it showed that the passive radon-thoron monitor used here is not affected by air humidity [10]. The vertical distributions of radon and thoron concentrations inside the accumulation chamber were obtained using a passive type radon-thoron discriminative monitor (RADUET, Radosys Ltd., Budapest, Hungary) [20]. The Raduets are composed of two different diffusion chambers of the same inner volume of about 30 cm^3^. The chambers are made of electroconductive plastic with a cylindrical form. The radon–thoron discrimination principle is based on the diffusion characteristics of each chamber. Radon in the air with its longer diffusion length is able to diffuse through an invisible air gap of one of the chambers located between its lid and bottom. Thoron can scarcely diffuse into that chamber with such a small pathway due to its very short half-life and lower diffusion length compared to that of radon. The second chamber has 6 holes of 6 mm of diameter opened at the side of the chamber which allow the diffusion of thoron as well as radon, the 6 holes are recovered by an electroconductive sponge to block the passage of charges particulate in the diffusion chamber [20]. The detection limits for the typical measurement period (3 months) were estimated to be 3 and 14 Bq m^−3^ for radon and thoron, respectively [21]. The RADUETs were placed at heights of 1, 3, 10, 30, and 80 cm from the ground surface inside the accumulation chamber. For laboratory analysis, the RADUETs were exchanged every three months: spring, March–May; summer, June–August; autumn, September–November; winter, December–February. The solid-state track detectors (CR-39; BARYOTRAK, Nagase Landauer, Ltd., Tsukuba, Japan), which were installed in the RADUETs, were taken out and chemically etched for 24 h in a 6M NaOH solution at 60 °C [21]. The number of alpha tracks was counted using an optical microscope and image analysis software (ImageJ, National Institutes of Health, Bethesda, Maryland, USA). Radon and thoron concentrations were calculated according to the International Organization for Standardization (ISO) 16641 [22]. The conversion factors from track densities of CR-39s to radon and thoron concentrations had been already evaluated using the radon-thoron calibration chamber in Hirosaki University [23]. Environmental parameters of temperature, relative humidity and atmospheric pressure inside the accumulation chamber were measured continuously using a portable type meteorological monitor (TR-73U, T&D Corp., Matsumoto, Japan).

### 2.2. Evaluation of Radon and Thoron Exhalation Rates Using the Ventilated Type Accumulation Chamber

According to Zhuo et al. [10], the radon exhalation rate *E*_Rn_ (mBq m^−2^ s^−1^) obtained using the ventilated type accumulation chamber can be calculated by Equation (1).

(1) $$E_{\mathrm{Rn}}=1000\times C_{\mathrm{Rn}}\times Z_{\max}\times \frac{Q+\lambda_{\mathrm{Rn}}}{3600}$$

Here, *C*_Rn_ is the average radon concentration at each height (Bq m^−3^), *Z*_max_ is the height of the chamber from the ground surface (=0.85 m), *Q* is air exchange rate in the accumulation chamber, and *λ*_Rn_ is the decay constant of radon (7.6 × 10^−3^ h^−1^). Air exchange rate in the chamber was evaluated using carbon dioxide (CO_2_) gas and a CO_2_ monitor (TR-76Ui, T&D Corporation). Generally, the air exchange rate is much larger than the decay constant of radon. Therefore, the decay constant of radon can be neglected (*Q* >> *λ*_Rn_, Q + *λ*_Rn_ ≈ *Q*).

According to the one-dimensional diffusion equation reported by Katase et al. [24], the thoron concentration *C*_Tn_(*z*) at a height *z* (m) above the ground is given by Equation (2).

(2) $$C_{\mathrm{Tn}}\left(Z\right)=\frac{E_{\mathrm{Tn}}}{\sqrt{\left(Q+\lambda_{\mathrm{Tn}}\right)D_{e}}}\cdot \exp\left(-\sqrt{\frac{Q+\lambda_{\mathrm{Tn}}}{D_{e}}}Z\right)$$

Here, *E*_Tn_ is the thoron exhalation rate from the ground (Bq m^−2^ s^−1^), *λ*_Tn_ is the decay constant of thoron (44.9 h^−1^), and *D*_e_ is the effective diffusion coefficient in free air (1.2 × 10^−5^ m^2^ s^−1^) [25]. In this study, the following exponential regression formula was applied to the vertical distribution of thoron concentration in each season for the simplified estimation of thoron concentration at 0 m.

(3) $$C_{\mathrm{Tn}}\left(Z\right)=a\cdot \exp\left(-bz\right)$$

Thoron concentration at the ground surface (*z* = 0 m) was estimated by Equation (3). Then, thoron exhalation rate *E*_Tn_ (mBq m^−2^ s^−1^) was evaluated by Equation (4) obtained by substituting *z* = 0 into Equation (2).

(4) $$E_{\mathrm{Tn}}=1000\times C_{\mathrm{Tn},0}\times \sqrt{D\cdot \frac{\left(Q+\lambda_{\mathrm{Tn}}\right)}{3600}}$$

Here, *C*_Tn,0_ is the thoron activity concentration at 0 m (Bq m^−3^).

### 2.3. Soil Parameters and Its Activity Concentrations of ^226^Ra and ^228^Ra 

Five soil core samples from the ground surface to 5 cm depth were collected using a stainless-steel soil sampler, which had a volume of 100 mL [26]. Dry bulk density, soil particle density, porosity, and soil textures were evaluated after drying samples for 24 h at 110 °C. The dry bulk density *ρ*_b_ was calculated as the mass of the dried soil divided by the soil volume. The soil particle density *ρ*_s_ was evaluated using a specific gravity bottle according to the test procedure of Japan Industrial Standards (JIS) A1202 [27]. The porosity ε was calculated by *ε* = 1−*ρ*_b_/*ρ*_s_. Soil particle size distribution was evaluated using a standard stainless-steel sieve for 0.075–2.0 mm particle size range and the sedimentation analysis for particles below 0.075 mm was made according to the test procedure of JIS A1204 [28] to determine the soil textures of the samples. The percentages of sand, silt, and clay for each soil sample were evaluated using the sample particle size distribution curves.

The activity concentrations of ^226^Ra and ^228^Ra were evaluated using a high-purity germanium semiconductor detector (GEM-100210, ORTEC, USA) with a relative efficiency of 30%, 1.85 keV energy resolution (FWHM) at 1.33 MeV of ^60^Co. The efficiency calibration of the detector was made using the standard volumetric sources which are contained ^109^Cd, ^57^Co, ^139^Ce, ^51^Cr^, 85^Sr, ^137^Cs, ^54^Mn, ^88^Y, and ^60^Co supplied by Japan Radioisotope Association. The detector was enclosed in a shielding made out of compacted lead of 10 cm of thickness. Each soil sample was enclosed in a cylindrical polypropylene container (U8 type container, 100 cm^3^) after drying for 24 h at 110 °C. The prepared soil sample was then enclosed in an air-tight container for 40 days to allow radioactive equilibrium between ^226^Ra and ^222^Rn to be reached. The measurement time was set as 80,000 s. In this study, the weighted average concentration of ^214^Pb and ^214^Bi were used as the ^226^Ra concentration in the soil samples by counting photons in the photoelectric peak channels of 352 keV for ^214^Pb and 609 keV for ^214^Bi. ^228^Ra was measured by counting photons in the photoelectric peak channel of 911 keV for ^228^Ac. The uncertainty for the activity concentration was evaluated taking into account the uncertainties of the counts for the sample and background. Coincidence summing, self-attenuation and decay corrections were applied using software (Gamma Studio, SEIKO EG&G, Tokyo, Japan).

### 2.4. Comparison of the Exhalation Rates with the Other Methods

#### 2.4.1. Accumulation Chamber with Scintillation Cell 

The stainless-steel accumulation chamber was set on the ground surface. Radon gas exhaled from the ground was accumulated for 1.5 to 3 h. Then, the radon gas inside the accumulation chamber was collected into a scintillation cell (Pylon 300A, Pylon Electrics, Inc., Toronto, Ontario Canada) at a sampling flow rate of 0.5 L min^−1^ and a sampling time of 5 min. After 3.5 h, the alpha counts from radon gas in the scintillation cell were measured using a portable radiation monitor (AB-5, Pylon Electrics, Inc., Toronto, Ontario Canada) [29]. The radon exhalation rate by grab sampling can be calculated by applying Equation (5).

(5) $$E_{\mathrm{Rn}}=\frac{\left(N-N_{b}\right)\cdot CF\cdot V\cdot \lambda_{\mathrm{Rn}}}{S\cdot \left[1-\exp\left(-\lambda_{\mathrm{Rn}}\cdot t\right)\right]}$$

Here, *N* and *N*_b_ are the count rates of the sample and background (cpm), *CF* is the conversion factor from count rate to radon concentration (27.0 Bq m^−3^ cpm^−1^) [30], *V* is the volume of the accumulation chamber (1.4 × 10^−2^ m^3^), *λ*_Rn_ is the decay constant of radon (2.1 × 10^−6^ s^−1^), *S* is the area under the accumulation chamber (9.9 × 10^−2^ m^2^), and *T* is the accumulation time (s).

#### 2.4.2. In Situ Radon and Thoron Exhalation Rate Monitor

Radon and thoron exhalation rates from the ground was also measured with an *in-situ* radon and thoron exhalation rate monitor (MSZ). The details of the method have been described by Saegusa et al. [31]. The monitor was composed of an accumulation chamber (volume, 13 L), a ZnS(Ag) scintillation detector with an aluminized mylar sheet, a light guide, a photomultiplier tube, a pulse counting part and scaler, and a timer. The area of an acrylic board coated with ZnS(Ag) scintillator was 0.12 m^2^. Count rates were recorded over consecutive 30-s intervals during a total recording period of 30 min after the monitor was set up on the ground. The conversion factors from count rates of 10 min and 30 min to exhalation rates of radon and thoron were 0.521 ± 0.040 mBq m^−2^ s^−1^ cpm^−1^ and 18.1 ± 3.2 mBq m^−2^ s^−1^ cpm^−1^, respectively. The measurement uncertainties for radon and thoron exhalation rates using the MSZ have been reported as ~20% and ~6%, respectively [11].

## 3. Results and Discussion

### 3.1. Physical Parameters of Soil and ^226^Ra and ^228^Ra Activity Concentrations at the Study Site

The percentages of sand, silt and clay for each soil sample collected at the study site were evaluated as 63 ± 4%, 16 ± 2%, and 21 ± 3%, respectively. As a result, the textural class of all soil samples was decided as sandy clay loam (SCL) based on a soil texture triangle. In general, the characteristics of SCL are reported to be high water retention and low air permeability [32]. Dry bulk density, soil particle density and porosity were evaluated as 1340 ± 19 kg m^−3^, 2657 ± 27 kg m^−3^, and 0.50 ± 0.07, respectively. These obtained values were not significantly different from the typical values of 1300−1350 kg m^−3^ for dry bulk density, 2600−2700 kg m^−3^ for soil particle density, and 0.3−0.6 for porosity [33]. Activity concentrations of ^226^Ra and ^228^Ra were evaluated to be 24.1 ± 0.4 Bq kg^−1^and 34.0 ± 0.9 Bq kg^−1^, respectively. According to the UNSCEAR [1], the Japanese mean activity concentrations of ^226^Ra and ^228^Ra (assuming radioactive equilibrium with ^232^Th) are reported as 33 Bq kg^−1^ and 28 Bq kg^−1^, respectively. Thus, radium activity concentrations in soil at the study site were found to be slightly lower than those of the national mean.

### 3.2. Radon and Thoron Concentration in the Ventilated-Type Accumulation Chamber

**Figure 2:**
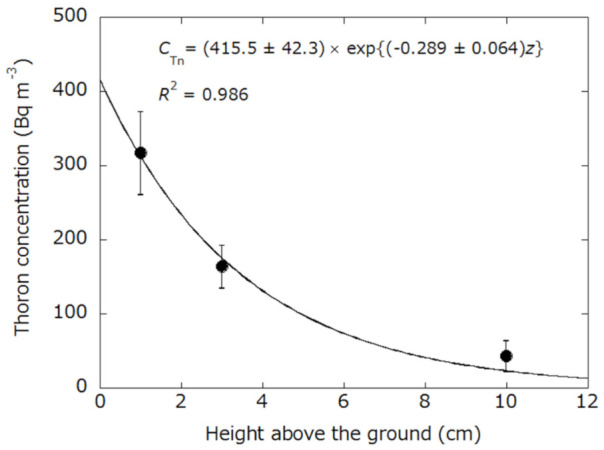
Example of the vertical distribution of thoron concentration inside the ventilated type accumulation chamber. The thoron concentration at ground level (*z* = 0 cm) was determined to be 416 ± 42 Bq m^−3^.

An example of the vertical distribution of thoron concentration inside the ventilated type accumulation chamber is shown in Figure 2. Thoron concentration decreased exponentially with the height above the ground surface. On the other hand, radon concentrations inside the accumulation chamber did not depend on the height above the ground. These observations were similar to the previously reported findings [16,17,19]. The results obtained at the 10 cm and above height from the ground were not considered in the calculation of thoron exhalation rate because the thoron concentrations at these heights were below the lower limit of detection. Additionally, the air exchange rate of the accumulation chamber was evaluated as 0.30 h^−1^ which was similar to the literature [9].

### 3.3. Comparison of Radon and Thoron Exhalation Rates Obtained by the Present System to Those Obtained by the Other Methods

Comparison of radon and thoron exhalation rates obtained by the ventilated-type accumulation chamber, accumulation chamber with scintillation cell and in situ monitor are shown in Table 1. Radon exhalation rates obtained by the passive method were in relatively good agreement with the results measured by the accumulation chamber with scintillation cell and in situ monitor (MSZ) taking into account the measurement uncertainty. Statistical analyses were conducted using the “EZR software” (Easy R) [34]. The difference was considered significant for *p* < 0.05. A one-way ANOVA test was performed for the comparison of radon exhalation rates obtained by each technique. Consequently, the radon exhalation rate measured by the present system and those obtained by the other methods are not significantly different (*p* = 0.128). Furthermore, the thoron exhalation rate obtained by the ventilated-type accumulation chamber in the first measurements was also in agreement with the result obtained by the in situ monitor (*p* = 0.156). However, in the second measurements, thoron exhalation rate obtained by the ventilated-type accumulation chamber was approximately half that of the value obtained by the in situ monitor. Theoretically, the diffusion length of thoron is reported as a few centimeters which is much shorter than that of radon due to the short half-life of thoron. Therefore, thoron exhalation rate is considered to be more strongly affected by the soil surface condition compared with the radon exhalation rate. Thus, it is necessary to make intercomparison experiments repeatedly to ensure the quality of the data obtained by the present ventilated-type accumulation chamber. However, the present method can offer an easy and low-cost system for the measurements of both radon and thoron exhalation rates, as no electric power supply is needed and operation and maintenance are easy.

### 3.4. Seasonal Variations of the Radon and Thoron Exhalation Rates

**Figure 3:**
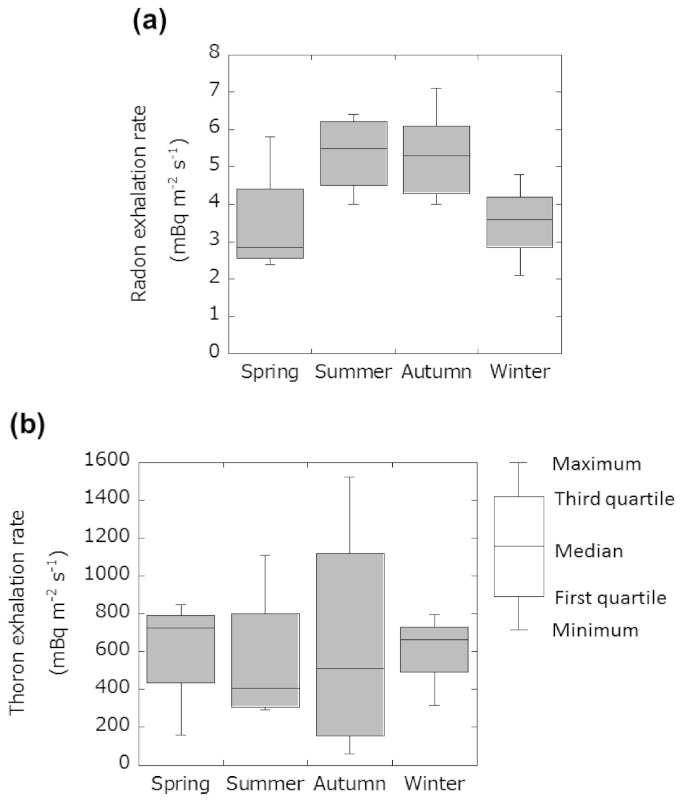
Seasonal variations of radon (**a**) and thoron (**b**) exhalation rates. The lines from top to bottom of the box-plot from top to bottom are defined as maximum, third quartile (75th percentile), median (50th percentile), first quartile (25th percentile), and minimum values.

Seasonal variations of radon and thoron exhalation rates are shown in Figure 3. The median values (range) of radon exhalation rate in spring, summer, autumn and winter were estimated to be 3.5 ± 0.5 (2.4−5.8), 5.4 ± 1.1 (4.0−6.4), 5.6 ± 0.3 (4.0−7.1), and 3.5 ± 0.3 (2.1−4.8) mBq m^−2^ s^−1^, respectively. The median values (range) of thoron exhalation rates were evaluated as 614 ± 126 (159−848), 555 ± 110 (295−1110), 563 ± 395 (61−1524), and 593 ± 138 (318−797) mBq m^−2^ s^−1^ for spring, summer, autumn and winter, respectively. Annual means of the radon and thoron exhalation rates were evaluated to be 4.5 ± 0.3 mBq m^−2^ s^−1^ and 581 ± 113 mBq m^−2^ s^−1^, respectively. According to the results of a large-scale survey in Japan [12,35], average radon and thoron exhalation rates from the ground were 8.6 mBq m^−2^ s^−1^ (*N* = 111) and 790 mBq m^−2^ s^−1^ (*N* = 405), respectively. Therefore, radon and thoron exhalation rates at the present measurement site were 52% and 74% of the Japanese averages. Furthermore, average radon exhalation rates in summer and autumn were higher than the annual mean. The ratio of the radon exhalation rate in summer to the radon exhalation rate in winter (or spring) is 1.5 and the ratio of the radon exhalation rate in autumn to the radon exhalation rate in winter (or spring) is 1.6. A one-way ANOVA was performed to determine whether there are any statistically significant differences between the means of each season. Consequently, it showed no statistically significant difference in the average radon exhalation rate between the seasons in this study (*p* = 0.103). However, the median values of radon exhalation rate tend to be higher from summer to autumn and lower from winter to spring (Figure 3a). Zhuo et al. [36] reported a similar seasonal variation of radon exhalation rate from the ground in China. Zhuo et al. [9] have also reported a negative correlation between radon exhalation rate and precipitation. On the other hand, Hosoda et al. [12] reported that when the variation of moisture saturation was small, the soil temperature appeared to induce a strong effect on the exhalation rate. However, when the variation of moisture saturation was large, the influence of moisture saturation appears to be larger than the soil surface temperature [12]. Furthermore, it has been also reported that an increase in the soil temperature markedly decreased the amount of adsorption of gases which contributed to the increase of emanation and diffusion coefficients [37,38]. Precipitation data from a location near the monitoring site were reported by the Japan Meteorological Agency [39], and cumulative precipitations in spring, summer, autumn, and winter during the measurement period were 424, 586, 441, and 177 mm, respectively. Additionally, their respective mean temperatures were 14.1, 25.2, 17.9, and 4.2 °C. Therefore, the radon exhalation rate in winter at the measurement site might be affected by low precipitation and temperature. On the other hand, in summer the high temperature might affect the radon exhalation rate.

The statistical analysis using the one-way ANOVA test showed that there was no significant difference between the averages of thoron exhalation rate for the different seasons (*p* = 0.982) (Figure 3b). According to the report by Prasad et al. [40], radon and thoron exhalation rates in summer and autumn were higher than those in spring and winter. However, the reported diffusion length of radon and thoron were a few meters and a few centimeters, respectively [11,26]. That is, thoron exhalation rate from the ground would be affected by such environmental parameters as moisture saturation and temperature around the surface soil. Therefore, the soil temperature and moisture saturation were measured continuously for three months in summer at 10 cm depth from the surface inside and outside of the accumulation chamber. The average surface soil temperature inside and outside the accumulation chamber were 22.3 ± 3.5 (RSD: 18%) and 22.2 ± 3.9 °C (RSD: 16%), respectively. The results suggested that the accumulation chamber setup was not affected by the surface soil temperature as same with the previous report [10]. On the other hand, the average moisture saturation (convert from volumetric water content using porosity) inside and outside the accumulation chamber were evaluated to be 0.364 ± 0.172 (RSD: 47%) and 0.114 ± 0.028 (RSD: 24%), respectively. As we mentioned above, Zhuo et al. reported that the water potential inside and outside of the stainless-steel box shown nearly the same throughout the year [10]. It is well known that the water potential is related parameter to volumetric water content which is influential parameter of exhalation rate. However, both the average moisture saturation and its variation inside the accumulation chamber were smaller than those outside the chamber. Additionally, the accumulation chamber was embedded 15 cm into the soil. That is, it is considered that this measurement condition was not easy for water due to rainfall to move from outside the chamber to under the chamber by passing through pore spaces in the surface soil. However, the thoron exhalation rates obtained in this study may be considered as baseline level at the measurement site. Thus, we will develop the correction method of thoron exhalation rates with the variation of the environmental factors. Additionally, it might be possible to evaluate the seasonal variations of thoron exhalation rate if a passive type radon and thoron discriminative monitor was set in a small size accumulation chamber.

## 4. Conclusions

In this study, radon and thoron exhalation rates from the ground were simultaneously evaluated for four years by applying the naturally ventilated accumulation chamber of a previous report. The results were compared to the data obtained by the accumulation chamber with scintillation cell and in situ radon and thoron exhalation rate monitor. The results of the present method had relatively good agreement with results of the other methods. Relationships between radon exhalation rates with environmental parameters were also observed and their variations with seasons were determined. The baseline level of thoron exhalation rate at the measurement site was evaluated. However, thoron exhalation rates did not show clear seasonal variations, most likely due to limitations of the present methodology. Therefore, the methodology will be modified based on the present results to allow the season variation of thoron exhalation rate from the ground to be obtained.

## Figures and Tables

**Table 1:** Comparison of radon and thoron exhalation rates obtained by the ventilated-type accumulation chamber, accumulation chamber with scintillation cell, and in situ monitor (MSZ) method.

Intercomparison	Methods	Radon Exhalation Rate (mBq m^−2^ s^−1^)	Thoron Exhalation Rate (mBq m^−2^ s^−1^)
1st measurement	Ventilated-type accumulation chamber	3.6 ± 0.5	797 ± 336
	Accumulation chamber with scintillation cell	4.6 ± 2.9	–
	In situ monitor (MSZ) method	1.1 ± 0.1	584 ± 103
2nd measurement	Ventilated type accumulation chamber	4.3 ± 0.5	1120 ± 321
	Accumulation chamber with scintillation cell	8.8 ± 5.3	–
	In situ monitor (MSZ) method	5.1 ± 1.8	474 ± 177

## Data Availability

Not applicable.
